# Cardiogenic Embolism Treated Successfully by Intravenous Recombinant Tissue-Type Plasminogen Activator Administration in a Young Child With Congenital Heart Disease: A Case Report

**DOI:** 10.1155/crpe/8259781

**Published:** 2025-11-29

**Authors:** Kengo Kurihara, Akira Saito, Shunya Hanakita, Satoshi Iihoshi, Soichi Oya

**Affiliations:** Department of Neurosurgery, Saitama Medical Center, Saitama Medical University, Saitama, Japan

**Keywords:** cerebral infarction, child, congenital heart disease, rt-PA administration, thrombolysis

## Abstract

Childhood ischemic stroke is rare. Although the standard treatment for ischemic stroke among adults is thrombolytic therapy with recombinant tissue-type plasminogen activator (rt-PA), its use in pediatric patients remains uncertain. Particularly, only a very few reports have studied this treatment among very young children aged < 3 years. We report the case of a 2.5-year-old boy with complex congenital heart disease who developed left hemiparesis and impaired consciousness. Magnetic resonance (MR) imaging confirmed an acute infarct in the right parieto-occipital lobe. He presented with severe neurological deficits and was treated with rt-PA at 3.5 h after symptom onset. The rt-PA at an adult dosage of 0.6 mg/kg was administered as an intravenous bolus and infusion. Initial computed tomography showed no hemorrhage. Post-treatment imaging showed recanalization and no new hemorrhage. The patient exhibited remarkable neurological improvement and was neurologically intact at 12 h postadministration. Follow-up MR imaging performed on Day 16 showed no evidence of ischemia. MR angiography clearly demonstrated the right parieto-occipital artery, which was not depicted on the initial MR images. Our case represents a rare instance of the use of rt-PA in a young child. Despite limited data and absence of pediatric-specific guidelines, this report suggests that rt-PA is effective and can be administered safely in young children with acute ischemic stroke, mirroring adult treatment protocols. However, further research is necessary to establish definitive pediatric guidelines.

## 1. Introduction

Childhood ischemic stroke is rare, occurring in only 2.7/100,000 people [1]. Its main cause is congenital heart disease, with a known frequency of 132/100,000 [2, 3].

Recently, with the advancements in neonatal and pediatric intensive care management and pediatric cardiac surgery, the survival rate of pediatric patients with congenital heart disease and number of cases of cardiogenic cerebral embolism have increased [4–6]. In adults, thrombolytic therapy with intravenous (IV) recombinant tissue-type plasminogen activator (rt-PA) is strongly recommended by the guidelines for acute stroke within 4.5 h of symptom onset [7–10]. Although some studies have reported that the IV administration of rt-PA among neonates and children is safe if it is performed for the correct indications under monitoring, there remain uncertainties concerning the specific considerations in its administration and the detailed treatment outcomes [11–15].

Herein, we report a case of acute ischemic stroke (AIS) in a 2-year-old boy with congenital heart disease that was effectively and safely treated with the IV administration of rt-PA.

## 2. Case Description

**Figure 1:**
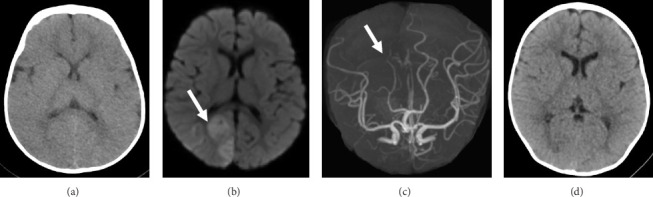
(a): An initial computed tomography (CT) scan image obtained on admission showing no obvious sign of infarction or hemorrhage. (b): A diffusion-weighted magnetic resonance (MR) image on admission demonstrating an acute infarction in the right occipital lobe (arrow). (c): MR angiogram revealing a right parieto-occipital artery occlusion (arrow). (d): A CT scan image on the day after rt-PA administration showing no obvious signs of hemorrhage or infarction.

**Figure 2:**
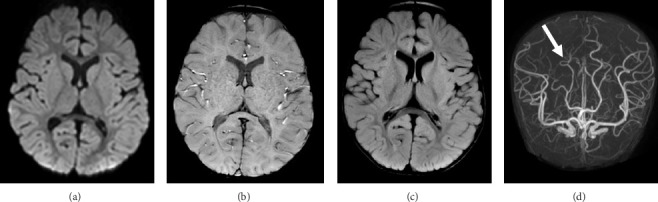
(a): An axial diffusion-weighted magnetic resonance (MR) image obtained at 16 days after stroke onset demonstrating the absence of an acute infarction. (b): An axial fluid-attenuated inversion recovery MR image depicting no radiological findings of infarction, even in the right parieto-occipital lobe. (c): An axial susceptibility-weighted MR image revealing no hemorrhagic lesion. (d): MR angiogram showing the recanalization of the occluded parieto-occipital artery.

A boy aged 2 years and 6 months with complicated congenital heart disease consisting of dilated cardiomyopathy, tricuspid valve dysplasia, and severe mitral regurgitation suddenly developed left hemiparesis and impaired consciousness. He was first taken to a nearby local hospital and urgently transferred to Saitama Medical Center, Saitama Medical University by a helicopter. On arrival, his blood pressure was 90/35 mmHg, his heart rate was 126 beats/minute with a sinus rhythm, and his oxygen saturation was 100% while receiving oxygen via a nasal cannula. He did not obey verbal commands although he could open his eyes. He also presented with left hemiparesis and right-side conjugate deviation. His pediatric National Institutes of Health Stroke Scale (NIHSS) score on arrival was 26. Head computed tomography (CT) scan showed no obvious intracranial hemorrhagic lesions (Figure 1(a)). Diffusion-weighted magnetic resonance (MR) imaging showed an acute infarct in the right parieto-occipital lobe (Figure 1(b)), and MR angiography showed an occlusion of the right parieto-occipital artery (Figure 1(c)). No other abnormal findings, including stenosis or occlusion of intracranial vessels, were observed. The laboratory tests showed anemia (hemoglobin, 7.9 g/dL), but no other coagulopathy, organ damage, elevated inflammatory response, or hypoglycemia was noted. Emergent transesophageal echocardiogram conducted by a pediatric cardiologist did not detect any intracardiac thrombus. At this point, 3.5 h had passed since stroke onset. According to the guidelines for adult patients, rt-PA administration is recommended within 4.5 h from stroke onset if there were no other contraindications [10]; however, the guidelines for pediatric patients with AIS are lacking. After a thorough discussion with the pediatric cardiologists, intensivists, and his parents, we then decided to perform IV administration of rt-PA. The dose of rt-PA given was the same as that for adults, specifically 0.6 mg/kg according to the Japanese guidelines [16]. As an IV bolus, 10% of the total dose (0.2 mL in our case) was administered over the span of 1 min and the remaining 90% of the dose was infused over the span of 60 min. Consequently, it was known at the time of stroke onset that the patient had no contraindications typically associated with adults, such as aortic disease. Neurological monitoring was performed every 6 h after the rt-PA administration. Obtaining accurate findings was more difficult in our pediatric patient than in adults because he required continuous sedation due to his congenital heart disease and unstable circulation. His blood pressure was 90/35 (mean 53) mmHg. He continued to take his regular antihypertensive medication after admission to reduce cardiac stress. As for the time course, IV rt-PA was administered at 3.5 h from stroke onset. Within an hour after treatment, the patient's right-side conjugate deviation disappeared and his paralysis improved. He obtained a pediatric NIHSS score of 10 on the day after rt-PA administration. CT scan performed on the following day revealed no indications of cerebral infarction (Figure 1(d)), even in the right parietal lobe that initially exhibited signs of acute cerebral infarction on the MR images at admission, suggesting recanalization of the infarcted area. The patient was considered neurologically intact at 12 h post-rt-PA administration. As an anticoagulation therapy, heparin (heparin 300 U IV to 150/kg/hour) was started at 24 h after the administration of rt-PA. Finally, he was switched from heparin to warfarin, and warfarin management was continued with a target prothrombin time-international normalized ratio (PT-INR) of 1.5–2.5 because of his poor cardiac function, as evidenced by a low left ventricular ejection fraction of 34%. At 16 days after stroke onset, no evidence of ischemia was noted by diffusion-weighted (Figure 2(a)) and fluid-attenuated inversion recovery MR imaging (Figure 2(b)). Susceptibility-weighted MR imaging revealed no hemorrhagic change (Figure 2(c)). MR angiography clearly demonstrated the right parieto-occipital artery, which was not depicted on the initial MR images (Figure 2(d)). There were no findings suggestive of hemorrhage in the susceptibility-weighted imaging either. The patient was discharged home with no neurological abnormalities at 19 days after admission. At the 9-month follow-up, no stroke or cardiac complications were observed, and the patient's weight has been steadily increasing. Blood coagulation test has been performed monthly to ensure that the PT-INR is within the optimum range. Currently, he is receiving 0.6 mg/day warfarin.

## 3. Discussion

Ischemic stroke rarely occurs in children. The precipitating risk factors for pediatric stroke include congenital heart disease, blood and coagulation system abnormalities, trauma, as well as moyamoya, arterial vascular, autoimmune, infectious, and metabolic diseases [4–6]. The incidence of pediatric stroke is increasing due to the advancements in neonatal and pediatric intensive care for severe congenital heart diseases [17]. However, a guideline similar to that in adults has not been established for pediatric patients because of the rarity of this condition [13].

In clinical practice, mechanical thrombectomy and rt-PA administration are implemented for pediatric acute stroke patients, as it is for adults [11–15, 18]; however, it is unclear whether rt-PA should be administered to children. The Thrombolysis in Pediatric Stroke study was a prospective study designed to evaluate the safety, optimal dosing, and feasibility of IV rt-PA therapy for pediatric AIS [19]. However, only a few of them ultimately received rt-PA, resulting in limited and inconclusive data. The reasons for the lack of clear guidelines for the administration of rt-PA to children are the lack of evidence of its safety and the small number of reported cases. The limited number of cases is likely attributable to the fact that, in many instances involving pediatric strokes, an accurate diagnosis cannot be established within 4.5 h of onset.

In various countries, guidelines regarding IV rt-PA administration for pediatric AIS differ based on the available evidence and expert consensus. In the United Kingdom, the use of rt-PA may be considered in children aged ≥ 8 years when AIS is clearly diagnosed [20]. For children aged 2 and 8 years, its administration should be carefully evaluated on a case-by-case basis. The eligibility criteria generally include symptom onset within 4.5 h, exclusion of intracranial hemorrhage by head CT, Pediatric NIHSS (pedNIHSS) score indicating at least moderate stroke severity, and the absence of bleeding disorders or other contraindicating comorbidities that constitute contraindications. In Australia, IV rt-PA is not considered a standard treatment for pediatric AIS, and its use is recommended only with extreme caution [21]. It may be considered only in highly selected cases that fulfill all of the following conditions: clearly defined acute onset of symptoms, exclusion of hemorrhage on neuroimaging, presence of a large-vessel occlusion, feasibility of treatment based on adult criteria, and availability of a multidisciplinary team with sufficient experience in pediatric stroke management. In Japan, the rt-PA administration is not generally recommended for pediatric patients under 18 years of age [22]. This cautious approach is based on the understanding that the pathophysiology and etiology of stroke in children differ significantly from those in adults, rendering the direct application of adult treatment protocols inappropriate. The Pharmaceuticals and Medical Devices Agency (PMDA) in Japan mentions that when rt-PA is considered for pediatric patients, it is generally administered according to adult guidelines, but the risks and benefits have not been established [23]. When rt-PA is considered to be potentially applicable, its administration should be considered only after careful evaluation and ideally within specialized centers equipped to manage such cases. Our hospital is formally designated as one of the tertiary pediatric emergency centers in Japan and has a multidisciplinary team comprising of pediatric cardiologists, intensivists, neurologists, and neurosurgeons experience in pediatric stroke management.

As for the safety, rt-PA administration may cause bleeding complications; however, in children, the incidence of bleeding complications is estimated to be 2%–4%, which is lower as compared to that in adults [24, 25]. The elevated rate of hemorrhagic complications observed in adults is likely due to the higher prevalence of risk factors, including elevated blood glucose levels, atrial fibrillation, renal impairment, chronic hypertension, and the use of antiplatelet medications in this population [25]. Recently, several case reports have demonstrated the safe and effective use of rt-PA in pediatric patients with acute cerebral infarction, but no clear guidelines have been established. In a series of 26 pediatric cases of AIS (age, 1.1–17 years) treated by IV rt-PA without endovascular therapy, 24 received rt-PA (0.9 mg/kg) within 2–4.5 h of stroke onset [25]. Among the remaining patients, one received a slightly increased dose of 0.99 mg/kg, whereas another was administered a reduced dose of 0.54 mg/kg (60% of the standard dose) due to parental refusal of a blood transfusion in case of hemorrhagic complications. No patient developed intracranial hemorrhage, and two patients had epistaxis. Although the number of cases is small, this study suggests that administering rt-PA as per adult criteria may be safe and effective in pediatric patients with AIS. However, reports of its use in young children aged < 3 years are extremely limited. Our literature search found only one case in which rt-PA was administered to a child aged ≤ 3 years. A 1-year-old boy developed left middle cerebral artery embolic occlusion secondary to congenital heart disease with an NIHSS score of 15. At slightly > 3.5 h after the onset of symptoms, rt-PA was administered at a dose of 0.9 mg/kg. Although subsequent follow-up CT did not reveal intracerebral hemorrhage, the assessment of cerebral infarction and vessel recanalization was not mentioned [25]. Our study is the first to provide the details of the clinical course, including MR imaging findings, following rt-PA administration in a young child aged < 3 years.

Based on the findings of the present case and the previously reported case, rt-PA may be used safely and effectively for AIS in young children following the same indication criteria and dosage as those in adults. Further case reports are needed to verify the safety of rt-PA administration for children.

## Data Availability

Data will be available upon reasonable request from the authors.

## Nomenclature

MR: Magnetic resonance
CT: Computed tomography
IV: Intravenous
NIHSS: National Institutes of Health Stroke Scale

## References

[1] Putaala J., Metso A. J., Metso T. M. (2009). Analysis of 1008 Consecutive Patients Aged 15 to 49 with first-ever Ischemic Stroke: the Helsinki Young Stroke Registry. *Stroke*.

[2] Hoffman J. L., Mack G. K., Minich L. L. (2011). Failure to Impact Prevalence of Arterial Ischemic Stroke in Pediatric Cardiac Patients over Three Decades. *Congenital Heart Disease*.

[3] Cheng H. H., Rajagopal S., McDavitt E. (2016). Stroke in Acquired and Congenital Heart Disease Patients and Its Relationship to Hospital Mortality and Lasting Neurologic Deficits. *Pediatric Critical Care Medicine*.

[4] Monagle P., Newall F., Barnes C. (2008). Arterial Thromboembolic Disease: a single-centre Case Series Study. *Journal of Paediatrics and Child Health*.

[5] Nasr D. M., Biller J., Rabinstein A. A. (2014). Use and in-hospital Outcomes of Recombinant Tissue Plasminogen Activator in Pediatric Arterial Ischemic Stroke Patients. *Pediatric Neurology*.

[6] Oren H., Devecioğlu O., Ertem M. (2004). Analysis of Pediatric Thrombotic Patients in Turkey. *Pediatric Hematology & Oncology*.

[7] Clark W. M., Wissman S., Albers G. W. (1999). Hamilton S: Recombinant Tissue-type Plasminogen Activator (Alteplase) for Ischemic Stroke 3 to 5 hours After Symptom Onset. the ATLANTIS Study: a Randomized Controlled Trial. Alteplase Thrombolysis for Acute Noninterventional Therapy in Ischemic Stroke. *JAMA*.

[8] Hacke W., Kaste M., Fieschi C. (1995). Intravenous Thrombolysis with Recombinant Tissue Plasminogen Activator for Acute Hemispheric Stroke. the European Cooperative Acute Stroke Study (ECASS). *JAMA*.

[9] Hacke W., Kaste M., Fieschi C. (1998). Randomised double-blind placebo-controlled Trial of Thrombolytic Therapy with Intravenous Alteplase in Acute Ischaemic Stroke (ECASS II). *The Lancet*.

[10] Emberson J., Lees K. R., Lyden P. (2014). Effect of Treatment Delay, Age, and Stroke Severity on the Effects of Intravenous Thrombolysis with Alteplase for Acute Ischaemic Stroke: a meta-analysis of Individual Patient Data from Randomised Trials. *The Lancet*.

[11] Kennedy L. A., Drummond W. H., Knight M. E., Millsaps M. M., Williams J. L. (1990). Successful Treatment of Neonatal Aortic Thrombosis with Tissue Plasminogen Activator. *The Journal of Pediatrics*.

[12] Newall F., Browne M., Savoia H., Campbell J., Barnes C., Monagle P. (2007). Assessing the Outcome of Systemic Tissue Plasminogen Activator for the Management of Venous and Arterial Thrombosis in Pediatrics. *Journal of Pediatric Hematology*.

[13] Evim M. S., Bostan Ö, Baytan B., Semizel E., Günes A. M. (2013). Thrombolysis with Recombinant Tissue Plasminogen Activator in 7 Children. *Clinical and Applied Thrombosis/Hemostasis*.

[14] Ansah D. A., Patel K. N., Montegna L., Nicholson G. T., Ehrlich A. C., Petit C. J. (2016). Tissue Plasminogen Activator Use in Children: Bleeding Complications and Thrombus Resolution. *The Journal of Pediatrics*.

[15] Tabone L., Mediamolle N., Bellesme C. (2017). Regional Pediatric Acute Stroke Protocol: Initial Experience During 3 Years and 13 Recanalization Treatments in Children. *Stroke*.

[16] Toyoda K., Koga M., Iguchi Y. (2019). Guidelines for Intravenous Thrombolysis (Recombinant Tissue-type Plasminogen Activator), the Third Edition, March 2019: a Guideline from the Japan Stroke Society. *Neurologia Medico-Chirurgica*.

[17] Oster M. E., Lee K. A., Honein M. A., Riehle-Colarusso T., Shin M., Correa A. (2013). Temporal Trends in Survival Among Infants with Critical Congenital Heart Defects. *Pediatrics*.

[18] Whiteley W. N., Slot K. B., Fernandes P., Sandercock P., Wardlaw J. (2012). Risk Factors for Intracranial Hemorrhage in Acute Ischemic Stroke Patients Treated with Recombinant Tissue Plasminogen Activator: a Systematic Review and meta-analysis of 55 Studies. *Stroke*.

[19] Rivkin M. J., deVeber G., Ichord R. N. (2015). Thrombolysis in Pediatric Stroke Study. *Stroke*.

[20] Royal College of Paediatrics and Child Health (2025). Stroke in Childhood: Clinical Guideline for Diagnosis, Management and Rehabilitation.

[21] Medley T. L., Miteff C., Andrews I. (2019). Australian Clinical Consensus Guideline: the Diagnosis and Acute Management of Childhood Stroke. *International Journal of Stroke*.

[22] Japanese Stroke Society (2023). *Guidelines for the Management of Stroke 2021 (Revised 2023)*.

[23] (2025). Information on the Use of rt-PA in Other Countries.

[24] (1995). Tissue Plasminogen Activator for Acute Ischemic Stroke. *New England Journal of Medicine*.

[25] Amlie-Lefond C., Shaw D. W. W., Cooper A. (2020). Risk of Intracranial Hemorrhage Following Intravenous tPA (Tissue-type Plasminogen Activator) for Acute Stroke Is Low in Children. *Stroke*.

